# Healthcare professionals feel empowered by implementing a hospital-based multifaceted intervention: a qualitative study using inductive thematic analysis

**DOI:** 10.1186/s12913-022-08310-w

**Published:** 2022-07-12

**Authors:** E. Klooster, N. Koenders, J. Vermeulen-Holsen, L. Vos, P. J. van der Wees, T. J. Hoogeboom

**Affiliations:** 1 Department of Rehabilitation, Deventer Hospital, Deventer, the Netherlands; 2 Radboud University Medical Center, Radboud Institute for Health Sciences, IQ Healthcare, Nijmegen, the Netherlands; 3 Radboud University Medical Center, Radboud Institute for Health Sciences, Department of Rehabilitation, Nijmegen, the Netherlands; 4 Erasmus Medical Center, Cardiovascular Institute, Rotterdam, the Netherlands; 5 Department of Psychology and Geriatrics, Deventer Hospital, Deventer, the Netherlands

**Keywords:** Qualitative research, Physical activity, Physical therapy, Hospitalization, Multifaceted intervention

## Abstract

**Background:**

Most patients are insufficiently physically active during their hospital stay, and this is associated with poor health and delayed recovery. Hospital-based multifaceted interventions aim to encourage patients to engage in physical activity. Ban Bedcentricity is one such intervention. Its value – and that of others similar to it – for healthcare professionals has not been studied yet. Whether an intervention looks and feels right, and whether it does the job well, is important for healthcare professionals and thus its use. Understanding value for healthcare professionals seems crucial for the long-term adoption and implementation of interventions. Therefore, we studied healthcare professionals’ perceptions of value in terms of the implementation of a multifaceted intervention that aimed at improving physically active behaviour in patients during their hospital stay.

**Methods:**

Using Ban Bedcentricity as a case study to focus on healthcare professionals’ perceptions about multifaceted interventions, we conducted a qualitative study between November 2019 and September 2020. Semi-structured interviews were conducted with purposefully selected physicians, physiotherapists, and nurses (assistants) until theoretical data saturation was reached. Inductive thematic analysis was used to identify key themes and develop a conceptual model.

**Results:**

We interviewed 15 healthcare professionals and formulated six key themes from these interviews. The participants said that Ban Bedcentricity empowered them in their beliefs about the importance of physical activity for hospitalized patients (theme 1). They also indicated that it made them more aware of the value of physical activity (theme 2) and skilled to promote physical activity as part of their professional role (theme 3). Similarly, they noted that it enabled them to shift from providing hands-on support to verbal coaching (theme 4). Other aspects that the participants valued were the increased possibilities for teamwork (theme 5) and the routinized physical activity promotion in usual care (theme 6). The challenges discussed by the participants were prioritizing activities that promoted physical activity, especially because of a high workload, and avoiding relapses of new routinized work practices related to physical activity promotion if insufficient long-term support and training were provided.

**Conclusions:**

Our conceptual model shows that the implementation of a hospital-based multifaceted intervention by healthcare professionals empowers their beliefs, and improves their awareness, skills, professional roles, teamwork, and work routinization. These values are typically overlooked, despite potentially being important facilitators for long-term implementation.

**Supplementary Information:**

The online version contains supplementary material available at 10.1186/s12913-022-08310-w.

## Introduction

The adverse effects of sedentary behaviour and physical inactivity in hospitalized patients have been described extensively [1–3]. Nevertheless, physical activity during hospitalization often proves challenging for patients [4–6]. In an attempt to reduce sedentary behaviour worldwide, healthcare professionals have developed and implemented several multifaceted interventions [7–9]. To understand the mechanisms of successful implementation, it is important to analyse healthcare professionals’ experiences with these kind of multifaceted interventions.

One of these multifaceted interventions is Ban Bedcentricity [10]. Ban Bedcentricity is an innovative program, which offers hospitals tools, action plans, and practical guidance. It aims to 1) improve the mindset, knowledge, and attitude of patients, their relatives, and healthcare professionals regarding the risks of sedentary behaviour and the benefits of physically active behaviour, 2) develop and provide adequate materials to support physical activity in patients during their hospitalization, and 3) improve the culture in hospitals regarding reducing sedentary behaviour and to promote physically active behaviour in patients [10]. Results after implementation of Ban Bedcentricity indicate favourable outcomes, including reducing sedentary behaviour and a proportionately greater number of patients being discharged home [10]. However, these outcomes do not guarantee successful, long-term implementation as there may be multiple barriers [11]. There are documented barriers to the promotion of physical activity promotion at the professional level, such as the belief that physical activity may result in injuries and the low prioritization of physical activity promotion [5]. Since multifaceted interventions are often developed, piloted, evaluated, and implemented by healthcare professionals, it seems important to be aware of what the value of such innovations is for healthcare professionals.

Value for healthcare professionals can be found on various levels. For example, healthcare professionals attribute value to innovations because of the impact on quality of care, patient safety, and costs [12]. If healthcare professionals are convinced that an innovation improves patient safety, this is a very powerful driver for culture change and the long-term implementation of an innovation [13]. Another important value is the reduction of workload [14]. A reduced workload frees up time for other professional activities, [15] lowers burnout risk, [16] and indirectly prevents staffing challenges [17]. Another more qualitative aspect is “promise”, which refers to whether an innovation “looks and feels right and does the job well” [18]. These kinds of qualitative values are crucial for early adoption [19].

More information about the value of multifaceted interventions that reduce sedentary behaviour in patients during their hospital stay for healthcare professionals could contribute to adoption and long-term implementation of promising interventions [20]. As such, we conducted a qualitative study that sought to answer the following research question: “What is the value of Ban Bedcentricity – a multifaceted intervention aimed at promoting physical activity among patients during their hospital stay – for physicians, physiotherapists, and nurses (assistants) from their own perspective?” An inductive thematic analysis was performed to provide an explanation for healthcare professionals’ experiences which were then processed in a conceptual model. Important aspects and themes were addressed, and new implementation insights were provided to improve adoption and long-term implementation.

## Methods

### Aim

This study aims to explain the perceived value of implementing a multifaceted intervention that aims to improve physically active behaviour in patients during hospital stays for healthcare professionals.

### Design

We conducted a qualitative study with an inductive, interpretative approach [21]. The purpose of using an inductive, interpretive approach was the systematic generation of theory from collected data [22]. We started with an area of interest—healthcare professionals’ perceptions of the multifaceted intervention Ban Bedcentricity – and collected and analysed data, and allowed relevant ideas to develop without preconceived theories that need to be tested for confirmation. This meant that we were able to construct the conceptual model as an interpretation of the healthcare professionals’ stories. The study protocol was approved by the Medical Research Ethics Committee CMO Radboudumc (registration number 2019–5861). Study reporting followed the Standards for Reporting Qualitative Research guideline (Additional file 1: Appendix 1) [23].

### Context and sample

The study was conducted at Radboud university medical center (Radboudumc) in Nijmegen, in the Netherlands, between November 2019 and September 2020. A purposeful sample of healthcare professionals was recruited who varied in age, gender, profession (i.e., nurse, physical therapist or physician (assistant)), and work experience. Inclusion criteria for the participants were: their age greater than 18 years and they had to have been working at one (or a combination) of the following hospital wards for six months or more: cardiothoracic surgery, cardiology, pulmonary diseases, orthopaedics and traumatology, neurosurgery, abdominal surgery, urology, obstetrics and gynaecology, oral- and maxillofacial surgery, cardiac care unit, or medical oncology. The participants were considered rich cases, as they were involved in the implementation of Ban Bedcentricity as an implementation officer or champion [10]. The implementation of Ban Bedcentricity was finalized at the participants’ hospital ward at the time of study sampling. We did not conduct any theoretical sampling, because the variation in age, gender, profession, and work experience directed our sampling strategy rather than new insights being gained into the developing theory. Sampling and data collection continued until no new conceptual insights were generated and the researchers confirmed theoretical saturation [21].

### Data collection

Data were collected through semi-structured interviews. The interviews were held face-to-face at the participant’s ward or online through video calls. Two clinical researchers (EK, NK) conducted the interviews, both of whom had a background in qualitative research. Three junior researchers (VvB, MP, MW) supported the data collection by providing technical support and observational memos. If relevant, observational memos were made during the interviews to record observations, interpretations, and behaviour.

An interview guide with open-ended questions was used to collect data on relevant topics, such as patients’ physical activity during hospital stay, the value of Ban Bedcentricity, and the adoption of Ban Bedcentricity (Additional file 2: Appendix 2). Probing questions were used to explore participants' thoughts, views, and perceptions of physical activity and Ban Bedcentricity. Prompting questions were then used to invite participants to explain the meaning and essence of the various topics (i.e., sedentary behaviour and physically active behaviour, the value of Ban Bedcentricity, and the adoption of Ban Bedcentricity).

### Data analysis

The audio of all the interviews was recorded and transcribed verbatim (EK, VvB, MP, MW). Data were analysed using inductive thematic analysis [24]. At all stages (open, axial, and selective coding), new data was compared with existing findings using the principles of constant comparison. First, interviews were analysed after which codes and themes were extrapolated from the data. By coding and categorising new interviews, the coding researchers (EK, JVH, and LV) compared existing codes and new categories and checked for fit with existing categories. Similarities and differences were raised and discussed with all authors. Theoretical saturation was deemed to be reached when categories were found to be dense and no new open codes could be extrapolated from the data [25]. ATLAS.ti (Scientific Software Development GmbH. Version 8.4, 2019) was used to support data analysis [26].

Data analysis for each interview was performed independently by two teams of clinical researchers (EK and JVH; or EK and LV). Transcripts were coded line-by-line using in-vivo coding (open coding). Open codes were then grouped into categories (axial coding). Preliminary themes were constructed (selective coding) concurrent to the open and axial coding of the transcripts. The selective codes were discussed in three meetings with all the coding authors (EK, JVH, and LV) until a consensus was reached on the key themes. By discussing the key themes (EK, JVH, and LV), a “bigger picture of the whole” was constructed, linking various categories. After this, key themes were presented to all authors and discussed in two consensus meetings. A preliminary theory was then constructed (EK), explaining the value of Ban Bedcentricity from the perspective of healthcare professionals. The preliminary theory was thoroughly discussed and improved in meetings with all the authors until a consensus was reached on the final conceptual model.

### Trustworthiness

Four quality criteria for trustworthy qualitative research were incorporated in the study design: credibility, dependability, confirmability, and transferability [27]. Credibility was improved using independent open coding by three clinical researchers (EK, JVH, LV) and, in subsequent stages, all authors performed collective analysis. Personal beliefs, judgments, and practices were critically assessed and bracketed at every stage of the data-analysis. There was some diversity in the research team’s expertise as two authors were involved in its implementation of Ban Bedcentricity (EK, NK), two authors were not involved in the implementation (JVH, LV), and two authors were involved for their research expertise with Ban Bedcentricity (PW, TH). A third party forward-back translated all quotes. Dependability was enhanced by using a member-checking technique. All participants received a written summary of the interview through email within five working days of the interview. Participants were asked to confirm or revise the summaries. The member-checking process improved the reporting of the findings as told by the participants themselves. To improve confirmability, we used starting questions to explore meanings and prompting questions to explain meanings. Because the participants were invited to explain things thoroughly, the authors were able to use the participants’ own language. To enhance the transferability of the data, the conceptual model was presented for external review to a group of junior researchers. The feedback of the reviewers was incorporated in the presentation of the conceptual model, however, the conceptual model itself was not changed.

## Results

We included the perspectives of 15 healthcare professionals. The characteristics of these participants are displayed in Table 1. The participants’ ages were between 24 and 62 years old, and most were female (12 out of 15) and nurses (10 out of 15). Their level of work experience ranged between 8 months and 39 years. The length of the interviews ranged between 17 and 46 min for face-to-face interviews (10 out of 15) and between 21 and 35 min for video interviews (5 out of 15), with an overall median length of 32 min. Theoretical saturation was reached after the eleventh interview. To confirm data saturation, four additional interviews were performed and analysed which did not reveal any new conceptual insights.

**Table 1:** Participants’ characteristics

Age in years, minimum—maximum	24 - 62
Female gender, percentage of participants (n)	80% (12)
Profession, percentage of participants (n)	
Physician	7% (1)
Physiotherapist	13% (2)
Nurse	67% (10)
Nurse assistant	13% (2)
Work experience in years, minimum—maximum	0.7 - 39

### Conceptual model

**Fig. 1:**
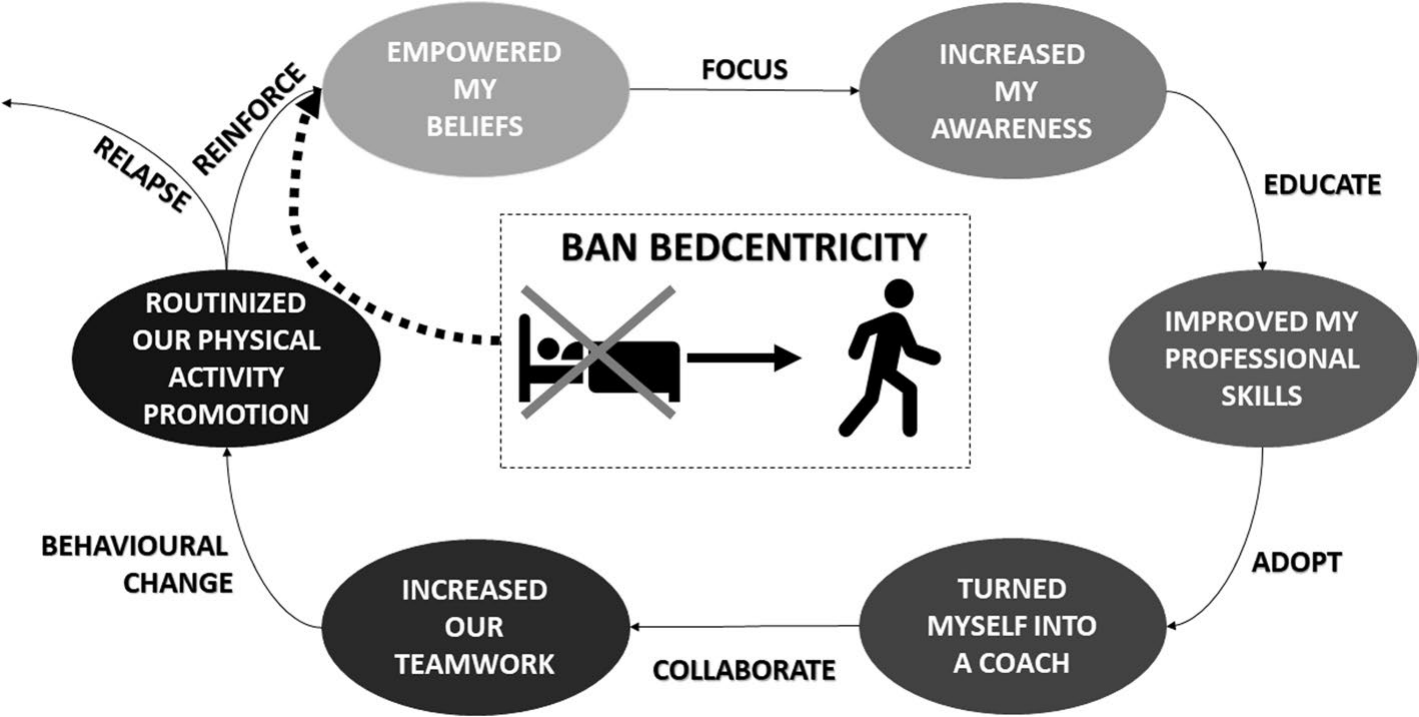
“Conceptual model of the value of Ban Bedcentricity for healthcare professionals. Healthcare professionals involved in the implementation of a hospital-based multifaceted physical activity intervention feel empowered in their beliefs, awareness, skills, coaching, teamwork, and work routines”

The study participants said that they perceived Ban Bedcentricity as being valuable for leading to behavioural change in themselves and their team. Its introduction at their hospital ward led to them behaving differently and resulted in their team routinely promoting physical activity. We identified six key themes and constructed a conceptual model to explain Ban Bedcentricity’s value from the healthcare professionals’ perspective (Fig. 1). We formulated six themes using the healthcare professionals’ perspective. The themes were formulated as follows. My involvement in the implementation of Ban Bedcentricity 1) empowered my beliefs, 2) increased my awareness, 3) improved my professional skills, 4) turned me into a coach, 5) increased our teamwork, and 6) routinized our promotion of physical activity.

The conceptual model starts with the idea that Ban Bedcentricity helped healthcare professionals to encourage patients from being sedentary to engaging physical activity. Healthcare professionals stated that Ban Bedcentricity empowered their beliefs that physically active behaviour is important for hospitalized patients’ health and well-being. A focus on promoting physical activity helped healthcare professionals to increase awareness and the sense of urgency surrounding physical activity as a means to improve health. Consequently, healthcare professionals appreciated education on how to promote physical activity. The Ban Bedcentricity team members then helped healthcare professionals to improve their professional skills (e.g., motivational interviewing). Their newly acquired skills helped them to adopt new professional roles and become coaches (i.e., transition from the role of a hands-on practitioner to that of a motivational coach). Subsequently, the healthcare professionals started to collaborate with colleagues, which enhanced their sense of working in a team. Ultimately, behavioural change at the healthcare professional level led to new habits for physical activity promotion that were routinized in day-to-day care practices. According to healthcare professionals, it was important to reinforce their habits regularly through education and to prevent relapse. The conceptual model represents a predominantly linear process, but starting and ending points may differ for healthcare professionals and their behavioural change can be completed in a cyclic-interactive manner.

### Ban Bedcentricity empowered my beliefs

The participants said that Ban Bedcentricity was a logical consequence of societal developments. They said that exercise is increasingly important for patients' health, and that patients should work on their recovery while in the hospital. Furthermore, the promotion of physical activity was considered as a social cause that they were eager to contribute to as part of their professional activities.

Pre-existing beliefs about the benefits of physical activity for hospitalized patients were very explicit among the participants. The participants believed it was important for patients to be physically active during their hospital stay, because it would contribute to both their mental and physical recovery, reduce complications (risks), and improve their overall well-being. Consequently, the participants believed in the concept of Ban Bedcentricity as a means to reduce unnecessary bed rest and to facilitate physically active behaviour during hospitalization, even before the program started at their ward. The healthcare professionals felt empowered in their beliefs, because Ban Bedcentricity emerged as an important program to get started with.

"You clearly see when people get out of bed more, when they walk around, that they recover quicker. And, often [they are] mentally so much more cheerful, cheerful is maybe a big word, but more talkative, able to process things a little faster. So, it is [important] not just for your condition and so on, but also for your mental state. That you also just enjoy moving around and being among people and not just lying in bed sick." (Participant 11936)

"There are really many reasons why it is important for patients to get out of bed, and I believe everyone here knows that and thinks it is an important thing here." (Participant 11918)

### The focus on physical activity improved my awareness

Ban Bedcentricity was not only valued because it related to pre-existing and new beliefs about physical activity held by healthcare professionals, but it also gave them the opportunity to focus on a single topic. Participants indicated that many healthcare innovations are implemented in quick succession and then quickly fade into oblivion. The supervised implementation of Ban Bedcentricity helped them to focus on the physically active behaviour of patients by not forgetting to support patients while dealing with day-to-day stressors, such as a high patient turnover and high workload.

"And that makes the patients realize that we think recovery is important and it is a good idea to put the focus on certain things." (Participant 37594)

The introduction of Ban Bedcentricity led to increased awareness among healthcare professionals of the promotion of physical activity. The participants said that information about the high amount of sedentary behaviour among their patients raised a sense of urgency. The participants started to reflect on their own roles in informing, teaching, and encouraging patients during their hospital stay.

"You are also very quickly tempted to go 'Oh, we'll do that tomorrow then...' or in a busy shift that you sometimes say - at least I notice that - that you then think 'Well that will be done tomorrow then'. But today in particular it is so important that someone gets out of bed, that you take a moment to do that, so I think for some it was the awareness that was very important." (Participant 65350)

At the same time, participants raised concerns about the risk of overloading patients. They mentioned “*a thin line in physical activity promotion between underloading and overloading patients*” (Participant 11,936). The participants talked about gaps in their own knowledge and insecurities about patients’ capacities, causing them to be insecure about their qualities for promoting physical activity. They questioned how they should monitor the limits of patients’ capacities and prevent overload.

"So that is something we as care providers do experience, that patients become overzealous and really want to do a lot at once. So, I notice that we must slow down patients sometimes, and give some extra information." (Participant 11899)

### Education improved my professional skills

The participants said that the increased awareness of the gaps in their knowledge initiated their call to be educated. They trained their physical activity promotion skills (e.g., motivational interviewing) and practiced with newly developed mobility promoting materials as part of the implementation of Ban Bedcentricity. In addition, they tested using the hospital ward as an attractive environment that promoted physical activity. The tests made participants feel heard and able to provide valuable input.

"It is really more conscious because of Ban Bedcentricity. That you start thinking more consciously of "Okay, but what can a patient do at this stage of admission? What can the patient do and then what can we do to support the patient in that?" (Participant 11899)

As the participants started to use Ban Bedcentricity principles during their day-to-day work, they began to question their patients’ physical capacity. With their newly acquired knowledge about the risk of under or overloading patients, the participants said they became less hesitant to use their new skills and support patients being physically active.

"Because we use motivational interviewing you know. Then I often say, "I have a lounge chair." They look at me. And then I take that and show it. And then I say, "Look, you can sit in it comfortably.” And then, oh yes, and then they sit in it... And then you will also hear “That is actually quite a comfortable chair”. It is nice to now have skills and knowledge to effectively engage patients.” (Participant 84525)

### I adopted my role and turned myself into a coach

However, the participants also said that promoting physical activity costs valuable time. They said that Ban Bedcentricity started their search to find a new balance between promoting physical activity and other important professional tasks. This time was not always available, and increased the participant’s need to adopt another professional role: that role of a coach.

"You know, it doesn't give me anything, but you do it for the patient. Because it does costs me time and energy to get that patient moving. But if I am motivated and know that this is supportive of the patient's healing process and rehabilitation, or to get through treatment well, that's the motivation. But when you say "What does Ban Bedcentricity do for you?” Nothing, it takes me time." (Participant 11590)

By offering less hands-on care and more verbal support, the participants said that they turned into coaches. By applying motivational interviewing techniques, patients were involved and tempted to make choices that improved their physically active behaviour. In addition, the participants said that they informed patients more frequently about the facilities available on the ward and in the hospital.

"I am more like... You need that and then I make sure that that process is set in motion. Yes, as it were coaching and being present afterwards. Because then you are looking at okay, the aspect of movement is important during your hospitalization, so I will start a conversation about that, about what do you need in this department?" (Participant 11590)

The participants also valued support from higher management staff, such as the hospital board of directors. They felt encouraged by their ward’s management in their implementation of Ban Bedcentricity and in acting as coaches. They noticed it was a hospital wide program implementation and felt connected to other implementation officers and champions. The participants said that it was “*not a one-person job*” (Participant 11027).

### Collaboration enhanced our teamwork

To fully fulfil their new coaching roles, healthcare professionals sought to collaborate with immediate colleagues, implementation officers, and champions. At this stage, the participants felt a clear shift from promoting physical activity as being the task of an individual, to the promotion of physical activity as being a team task. Working as a team felt good and ensured that real change took place.

“But because I was really on top of things together with [name], and we implemented all kinds of changes, other people also got motivated and they also liked to see that the things they brought up, that something actually happened with it in our team.” (Participant 75221)

To change patients’ attitudes towards physical activity, the participants said they needed to work as a team and adopt Ban Bedcentricity throughout the entire hospital. This would make patient care more efficient and ultimately create a more pleasant working environment. According to the participants, not all colleagues immediately took part in the team work; some were a bit hesitant, however, in the end, it was felt that teamwork was necessary to jointly promote physical activity in patients during their hospital stay.

“Awareness also among the team and colleagues, but some colleagues were a bit hesitant, a bit reluctant and this program also supports them to approach that a bit more loosely, to get the patient moving more.” (Participant 11027)

### We changed our behaviour and routinely promoted physical activity

The participants said they saw a need for behavioural change. This time, it was not a need for behavioural change among patients, but behavioural change in themselves. It was noticed that some team members changed their behaviour quicker than others.

“And I've learned here that that behavioural change... that you just have to take time for it. Because not everything that changes makes everybody happy, and this takes a long time. You can move forward as an individual, but the rest also has to get there and that sometimes takes a few years.” (Participant 75221)

Ultimately, the participants routinized Ban Bedcentricity in a way that worked for their team. While some participants stated that the program did not make a substantial difference, other participants said that Ban Bedcentricity strengthened their beliefs about and awareness of promoting physical activity. For some participants, it seemed that Ban Bedcentricity had become part of usual care, which resulted in its integration to such an extent that participants found it difficult to identify what changes could be contributed to Ban Bedcentricity and what could not.

“I don't think it has changed my work in such a way that I think 'Oh dear'. You're more aware, that's mainly it. It has been routinized suddenly. I think that was also just the strength of the implementation process. That it all crept in. That it wasn't a complete turnaround all at once, but that it really just step-by-step integration…” (Participant 11899).

For the long-term promotion of physical activity, the participants emphasized the importance of reinforcement by repeating the educational programs and following new developments. Boostering Ban Bedcentricity sessions could help to maintain the newly routinized physical activity promotion, according to the participants. They feared relapse if the reinforcements were lacking or not secured through protocol. Participants also said that it might be important to continuously involve so-called “late adaptors” and “laggards”, so they did not influence others’ enthusiasm. Overall, the participants indicated that they felt very empowered in their beliefs about the importance of physically active behaviour and that they would never relinquish them.

“But what I find difficult in any case in our department, where I am still searching, is when new things are introduced, to hold on to that, to keep it warm. I notice that with everything actually, it quicky fades away. And that's also the case with Ban Bedcentricity, so that has not so much to do with [names], but simply with the culture or attitude that prevails in our department. And it is really very important to have all the new things, new agreements, to just continually repeat them and keep them warm and discuss them. And really have a support base. And yeah, I don't know so well, how we should… How to change that. We are still working on that.” (Participant 75221)

## Discussion

This qualitative study explains the value of a multifaceted intervention that reduces sedentary behaviour in hospitalized patients for healthcare professionals according to the perspectives of healthcare professionals themselves. We identified six key themes and constructed a conceptual model (Fig. 1). The participants said that Ban Bedcentricity empowered their beliefs about the importance of physical activity for hospitalized patients. they also indicated that it made them more aware of and skilled in promoting physical activity as part of their professional role – shifting from providing hands-on support to verbal coaching. Other aspects that the participants valued included the increased possibilities for teamwork and routinized physical activity promotion in usual care.

We found many similarities when comparing our results with those of previously published studies by King, [28] Zisberg [29] and Pedersen [30]. King et al. [28] developed a program called “Mobilizing Older adult patients VIa a Nurse-driven intervention” (MOVIN). They found three central categories (shifting ownership, feeling supported, and making ambulation visible) that described the effect of MOVIN on nursing staff behaviours and perceptions of the intervention. The category "shifting ownership" was very similar to our conceptual model, where focus, education, adoption, and collaboration ultimately transferred the ownership of physical activity stimulation from individual professionals to entire teams of healthcare professionals. Zisberg et al. [29] created a multifaceted intervention called "WALK-FOR" to reduce barriers, re-shape staff attitudes and knowledge, and increase in-hospital mobility among older adults. Our conceptual model described how the implementation of Ban Bedcentricity influenced attitudes, knowledge, and behaviour of healthcare professionals. Both interventions were multifaceted and ultimately succeeded in behavioural change of entire teams of healthcare professionals. Pedersen et al. [30] designed the WALK-Copenhagen intervention. Their interviews with physicians showed that facilitators for encouraging mobility were enhanced by cross-professional cooperation that focused on mobility, physician encouragement of mobility and patient independence ( e.g., in picking up beverages and clothes) [31]. Our conceptual model also showed that focus, encouragement and targeting multiple levels were crucial aspects for Ban Bedcentricity’s value for healthcare professionals.

The themes we present are also in line with earlier research that explores healthcare professionals’ perspectives on physical activity promotion in patients during hospital stay. Chan et al. [32] and Kneafsey et al. [33] state that nurses see physical activity promotion as a “fundamental facet of nursing”. Nurses find it essential to promote physically active behaviour and thus contribute to the prevention of functional decline. In our study, we show that participants felt empowered in their beliefs and professional role in promoting physically active behaviour by Ban Bedcentricity. This is important, because alignment with pre-existing beliefs contributes to adoption and the long-term implementation of interventions [11]. Chan et al. [32] also present “knowing patients’ functional ability” as a healthcare professional-based facilitator for the promotion of physical activity. Our study shows that Ban Bedcentricity increased awareness of participants of their gaps in knowledge related to a thin line in physical activity promotion between underloading and overloading patients. The participants value education, training, practicing, and the testing of Ban Bedcentricity activities (e.g., motivational interviewing) for effective physical activity promotion. Avancini et al. [34] present the importance of a "*teamwork approach*", where nurses work with each other and with an exercise specialist to deploy personalized physical activity programs and provide resources such as booklets and pamphlets to patients. In our study, participants talked about the value of Ban Bedcentricity for increasing collaboration between healthcare professionals which is very similar. For adoption and long-term implementation, this means the participants value networks and communication with others.

The values expressed by the participants correspond to constructs in the Consolidated Framework for Implementation Research [11]. This framework provides a set of five constructs associated with the effective implementation of multifaceted interventions: individuals involved, inner setting, outer setting, tailored intervention, and implementation process [11]. When we compare our key themes and conceptual model to the constructs of the Consolidated Framework for Implementation Research, we note that our conceptual model provides little information on the 'outer setting'. Our focus was on implementation within our organization, although more information about the outer setting (i.e., external stakeholders’ perspectives, regulatory measures, and financial arrangements) may be useful for the adoption and long-term (national) implementation of Ban Bedcentricity.

We notice that our conceptual model illustrates the routinization of physical activity promotion from a predominantly positive and linear perspective. This may be the result of the research aim (i.e., value is a positive construct, as opposed to a challenge or barrier) and our study sample (i.e., rich cases were involved in Ban Bedcentricity, and they may have been more positive about the promotion of physical activity by nature). However, we are aware of the importance of dealing with barriers to the adoption and long-term implementation of multifaceted interventions. In our study, we show that healthcare professionals talked about the challenges of prioritizing physical activity promotion and achieving behavioural change of patients. These challenges are in line with known barriers to healthcare professionals. Geelen et al. [5] state that physical activity promotion receives a lower priority than medical treatment or (bed) rest as a result of high workload and safety. They also mention that prompting behavioural change among patients is difficult due to the”passive and dependent attitude patients adopt during hospitalization”. Overall, the priority of patient safety precautions appears to be the overarching barrier the promotion of physical activity in hospital care [35, 36]. This excessive culture of safety in hospitals is the multifaceted phenomenon that multifaceted interventions – such as Ban Bedcentricity – challenge while never losing sight of patient safety.

### Strengths and limitations

Qualitative research methodologies rely on the trustworthiness of the process and theory [37]. The rigor of this study is the result of the methodology, which is consistent with the research question. Inductive thematic analysis was used to explain healthcare professionals’ perspectives on the promotion of physical activity as part of the multifaceted intervention, Ban Bedcentricity. This study generated rich, explanatory data from a specific participant group sampled to theoretical saturation. Although the perspectives of a small group may seem vulnerable, credible data collection and analysis ensured that the results can be viewed with confidence and interpreted in a trustworthy manner. The transferability of the conceptual model outside of promoting physical activity in hospitals may not seem appropriate, however, the values explained in this study can inspire the implementation of all kinds of multifaceted interventions.

As well as there being strengths, there are also some limitations. First, we did not follow all principles of grounded theory such as theoretical sampling and the iterative construction of the interview guide(s). Not following methodological principles closely can result in method slurring [22]. Unlike our sampling, which was planned beforehand, theoretical sampling in grounded theory continues throughout the study and should not be planned before the study starts. We sampled our participants based on a-priori characteristics such as age, gender, profession, and work experience. This led to the inclusion of rich cases in our study. However, theoretical sampling could have been applied to search for ideas that confirmed or enriched our conceptual model. Our interview guide was prepared in advance, but the interviewers used unreported prompting and probing questions in follow-up interviews, as is customary in semi-structured interviews. An extension of methods would likely have led to an even better understanding of the research question. No negative cases were sampled in this study. Negative cases, also known as deviant cases or outliers, are cases in which participants’ viewpoints differ or seem not to support the main conceptual model [21]. In this study, negative cases could have nuanced or questioned the value of Ban Bedcentricity for physical activity promotion among healthcare professionals, There are some disadvantages of Ban Bedcentricity to healthcare professionals mentioned in the current study, such as the necessary time investment and the risk relapse; however, the sample of individuals involved in Ban Bedcentricity may have led to predominantly positive views. In addition, data triangulation with participating observations may have improved our understanding of the healthcare professionals’ perspectives. Participating observations can be performed by a researcher studying a group not only by observing the group, but also by participating in the activities of the group (in this case: healthcare professionals) [21]. Values can sometimes be difficult to express in language during an interview, but they can be spontaneously discussed during work. Data triangulation with participating observations could therefore have led to new conceptual insights. Lastly, the conceptual model appears to be most fitting for nurses, but less fitting for physicians. This has to do with the shifting professional role from 'hands-on' to being a 'coach', a finding that physiotherapists and nurses (assistants) recognized themselves in.

## Conclusion

Our conceptual model explains that the implementation of a hospital-based multifaceted intervention for physical activity promotion by healthcare professionals empowered their beliefs, awareness, skills, professional roles, teamwork, and work routinization. Insight into these values for healthcare professionals themselves contributes to culture change and the long-term implementation of an innovation. These values associated with implementing multifaceted interventions are typically overlooked, while they may be important facilitators for long-term implementation.

## Supplementary Information

**Additional file 1:** **Appendix 1.** Standars for Reporting Qualitative Research (SRQR).

**Additional file 2:** **Appendix 2.** Interviewguide.

## Data Availability

The datasets generated and analysed during the current study are not publicly available because it is practically impossible to completely anonymize all audio materials and transcripts. However, anonymized data are available from the corresponding author on reasonable request.

## References

[1] Brown CJ, Redden DT, Flood KL, Allman RM (2009). The underrecognized epidemic of low mobility during hospitalization of older adults. J Am Geriatr Soc.

[2] Covinsky KE, Pierluissi E, Johnston CB. Hospitalization-associated disability:“She was probably able to ambulate, but I’m not sure.” JAMA. 2011;306(16):1782–93. 10.1001/jama.2011.1556 22028354

[3] Lafont C, Gérard S, Voisin T, Pahor M, Vellas B (2011). Reducing, “iatrogenic disability” in the hospitalized frail elderly. J Nutr Health Aging.

[4] Fazio S, Stocking J, Kuhn B, Doroy A, Blackmon E, Young HM (2020). How much do hospitalized adults move? A systematic review and meta-analysis. Appl Nurs Res.

[5] Geelen SJG, van Dijk-Huisman HC, de Bie RA, Veenhof C, Engelbert R, van der Schaaf M (2021). Barriers and enablers to physical activity in patients during hospital stay: a scoping review. Syst Rev.

[6] Koenders N, Marcellis L, Nijhuis-van der Sanden MW, Satink T, Hoogeboom TJ (2021). Multifaceted interventions are required to improve physical activity behaviour in hospital care: a meta-ethnographic synthesis of qualitative research. J Physiother.

[7] Cohen Y, Zisberg A, Chayat Y, Gur-Yaish N, Gil E, Levin C (2019). Walking for better outcomes and recovery: the effect of WALK-FOR in preventing hospital-associated functional decline among older adults. J Gerontol Series A.

[8] Liu B, Moore JE, Almaawiy U, Chan W-H, Khan S, Ewusie J (2018). Outcomes of Mobilisation of Vulnerable Elders in Ontario (MOVE ON): a multisite interrupted time series evaluation of an implementation intervention to increase patient mobilisation. Age Ageing.

[9] van Delft LM, Bor P, Valkenet K, Slooter AJ, Veenhof C (2020). The effectiveness of Hospital in Motion, a multidimensional implementation project to improve patients’ movement behavior during hospitalization. Phys Ther.

[10] Koenders N, Potkamp-Kloppers S, Geurts Y, Akkermans R, Nijhuis-van der Sanden MW, Hoogeboom TJ (2021). Ban Bedcentricity: a multifaceted innovation to reduce sedentary behavior of patients during the hospital stay. Phys Ther.

[11] Damschroder LJ, Lowery JC (2013). Evaluation of a large-scale weight management program using the consolidated framework for implementation research (CFIR). Implement Sci.

[12] Yong PL, Olsen L, McGinnis JM (2010). Value in health care: accounting for cost, quality, safety, outcomes, and innovation.

[13] Tucker SJ, Carr LJ (2016). Translating physical activity evidence to hospital settings: a call for culture change. Clin Nurse Spec.

[14] Fishbein D, Nambiar S, McKenzie K, Mayorga M, Miller K, Tran K (2019). Objective measures of workload in healthcare: a narrative review. Int J Health Care Qual Assur.

[15] Yanchus NJ, Ohler L, Crowe E, Teclaw R, Osatuke K. ‘You just can’t do it all’: a secondary analysis of nurses’ perceptions of teamwork, staffing and workload. J Res Nurs. 2017;22(4):313–25.

[16] Portoghese I, Galletta M, Coppola RC, Finco G, Campagna M (2014). Burnout and workload among health care workers: the moderating role of job control. Saf Health Work.

[17] Duffield C, O’Brien-Pallas L. The causes and consequences of nursing shortages: a helicopter view of the research. Aust Health Rev. 2003;26(1):186–93. 10.1071/ah030186 15485390

[18] Campbell B (2012). How to judge the value of innovation. BMJ.

[19] Bienkowska-Gibbs T, Exley J, Saunders CL, Marjanovic S, Chataway J, MacLure C (2016). Evaluating the role and contribution of innovation to health and wealth in the UK: a review of innovation, health and wealth: phase 1 final report. Rand Health Q.

[20] Lu AD, Kaul B, Reichert J, Kilbourne AM, Sarmiento KF, Whooley MA (2021). Implementation strategies for frontline healthcare professionals: people, process mapping, and problem solving. J Gen Intern Med.

[21] Corbin J, Strauss A (2014). Basics of qualitative research: Techniques and procedures for developing grounded theory: Sage publications.

[22] Holloway I, Galvin K. Qualitative research in nursing and healthcare. Hoboken: John Wiley & Sons; 2016.

[23] O’Brien BC, Harris IB, Beckman TJ, Reed DA, Cook DA (2014). Standards for reporting qualitative research: a synthesis of recommendations. Acad Med.

[24] Chapman A, Hadfield M, Chapman C (2015). Qualitative research in healthcare: an introduction to grounded theory using thematic analysis. J R Coll Physicians Edinb.

[25] Charmaz K (2008). Grounded theory as an emergent method. Handbook of emergent methods.

[26] Friese S. Qualitative data analysis with ATLAS. ti. Thousand Oaks: Sage; 2019.

[27] Steinke I (2004). Quality criteria in qualitative research. A Companion to Qualitative Research.

[28] King BJ, Steege LM, Winsor K, VanDenbergh S, Brown CJ (2016). Getting patients walking: a pilot study of mobilizing older adult patients via a nurse-driven intervention. J Am Geriatr Soc.

[29] Zisberg A, Agmon M, Gur-Yaish N, Rand D, Hayat Y, Gil E (2018). No one size fits all—the development of a theory-driven intervention to increase in-hospital mobility: the “WALK-FOR” study. BMC Geriatr.

[30] Pedersen BS, Kirk JW, Olesen MK, Grønfeldt BM, Stefánsdóttir NT, Brødsgaard R (2022). Feasibility and implementation fidelity of a co-designed intervention to promote in-hospital mobility among older medical patients—the WALK-Copenhagen project (WALK-Cph). Pilot Feasibility Stud.

[31] Stefánsdóttir NÞ, Pedersen MM, Tjørnhøj-Thomsen T, Kirk JW (2021). Older medical patients’ experiences with mobility during hospitalization and the WALK-Copenhagen (WALK-Cph) intervention: a qualitative study in Denmark. Geriatr Nurs.

[32] Chan E-Y, Hong MLI, Tan MY-HG, Chua W-L (2019). Older patients’ participation in physical activity during hospitalization: a qualitative study of ward nurses’ perceptions in an Asian context. Geriatric Nursing.

[33] Kneafsey R, Clifford C, Greenfield S (2013). What is the nursing team involvement in maintaining and promoting the mobility of older adults in hospital? A grounded theory study. Int J Nurs Stud.

[34] Avancini A, D’Amico F, Tregnago D, Trestini I, Belluomini L, Vincenzi S, et al. Nurses perspectives on physical activity promotion in cancer patients: a qualitative research. Eur J Oncol Nurs. 2021;55:102061. 10.1016/j.ejon.2021.102061 34763207

[35] Singer SJ, Tucker AL (2005). Creating a culture of safety in hospitals. Abstr Academy Health Meet.

[36] Resnick B, Galik E, Wells CL, Boltz M, Holtzman L (2015). Optimizing physical activity among older adults post trauma: Overcoming system and patient challenges. Int J Orthop Trauma Nurs.

[37] Nicholls D (2017). Qualitative research. Part 2: methodologies. Int J Ther Rehab.

