# Supplementary material for: Healthcare professionals feel empowered by implementing a hospital-based multifaceted intervention: a qualitative study using inductive thematic analysis
Source: BMC Health Serv Res. 2022 Jul 12;22:903. doi: 10.1186/s12913-022-08310-w (PMC9277783; doi:10.1186/s12913-022-08310-w)
Supplement: Supplementary file 2 — Additional file 2: Appendix 2. Interviewguide. [file 12913_2022_8310_MOESM2_ESM.docx]

# APPENDIX 2. INTERVIEWGUIDE

Introduction

The aim of this study is to understand the value Ban Bedcentricity from the perspective of health professionals. Your input during the interview will help us with this research. The interview will last a maximum of 45 minutes. First, we will ask some questions about sedentary behaviour and physically active behaviour in general, followed by a few questions about the Ban Bedcentricity intervention. I would like to ask you to answer the questions as extensively as possible, this helps us to understand your opinion and thoughts. It helps us if you think out loud, because then your answers will be on the audio recording and we can use it for our research. Every answer is right and every opinion counts.

Topic: Sedentary behaviour and physically active behaviour of patients during their hospital stay

I would like to first ask a few questions about sedentary behaviour and physically active behaviour in general.

| 1. What are your first thoughts about sedentary behaviour of patients at your hospital ward? | What are important aspects of sedentary behaviour for patients during their hospital stay? Can you tell me more about that? |
| --- | --- |
| 1. What are your first thoughts about physically active behaviour of patients at your hospital ward? | What are important aspects of physically active behaviour? Please elaborate. |
| 1. What does it mean for your work as a healthcare professional when patients are physically active? | Can you describe a normal workday in which you are supporting patients to be physically active? |
| → Prompting on illustrative examples and professional activities. | Is it important for you to support patients to be physically active? Can you tell me more about that? |
|  | How do you support patients to be physically active? What professional activities do you perform daily? |
| 1. What do you think are the advantages and disadvantages for your work as a healthcare professional when patients are physically active? | Can you give an example that illustrates this? Why was this an advantage or disadvantage? |

Topic: Value of Ban Bedcentricity

For this topic, I will ask some questions about the value of Ban Bedcentricity from your perspective. Please let me know your opinions and thoughts.

| 1. What do you think is the influence of Ban Bedcentricity on the sedentary behaviour and physically active behaviour of patients? | Why do you think this way? Are there illustrative examples that support your thoughts? |
| --- | --- |
| 1. What do you think is the influence of Ban Bedcentricity on your work? | Why do you think this way? Can you tell me more about that? |
| → Prompting on costs, quality of care, patient safety, and promise | Is there something that you value about Ban Bedcentricity? Please elaborate |
| 1. What is your opinion on the Ban Bedcentricity intervention? | What is your overall view on the Ban Bedcentricity intervention? |
| → Prompting on professional activities | Which tasks were easier or more difficult? Why? |
| 1. What do you think of the changes in your work as a result of the Ban Bedcentricity intervention? | Have there been significant changes in your tasks? Why are these especially important to you? |

Topic: Adoption of Ban Bedcentricity

We have now talked about the value of Ban Bedcentricity for you as a healthcare professional. The following topic focusses on what Ban Bedcentricity means for your day-to-day practice.

| 1. How do you use Ban Bedcentricity in your day-to-day practice? | What helps you to perform professional activities in line with Ban Bedcentricity? Why does this help? |
| --- | --- |
|  | What are the advantages or disadvantages of Ban Bedcentricity for your daily work? |
| 1. How did you adopt Ban Bedcentricity in your day-to-day practice? | What did help you to use Ban Bedcentricity in your daily work? |
| → Prompting on skills training (i.e. motivational interviewing), practicing with new materials and testing the new hospital environment | What are the advantages or disadvantages now you use Ban Bedcentricity? |
| 1. What do you need to be able to better apply Ban Bedcentricity during your work? | What do you think contributed to being able to apply Ban Bedcentricity during your work? |
| → Prompting on differences between before and after implementation | How did you apply physical activity promotion before implementation of Ban Bedcentricity? Has anything changed? |
| 1. What do you need to make Ban Bedcentricity better match your work? | Are there things that made Ban Bedcentricity a good fit for your daily work? And were there things that absolutely did not fit? |
| → Prompting on differences between before and after implementation | How did physical activity promotion fit you’re your daily work before implementation of Ban Bedcentricity? Has anything changed? |

Characteristics

I would like to record your personal data. Therefore, we will end with a few close-ended questions:

- What is your name?
- What is your date of birth?
- What is your gender?
- What is your profession?
- What is your highest level of education?
- What hospital ward do you work in? (number and name)
- How long have you been working at the hospital ward?
- How long have you been working with Ban Bedcentricity?

Closing

Do you currently have any questions about the interview? We want to call you in two days to give a summary of this interview. We do this to check whether we have correctly understood what you have told in this interview. You can respond to our summary during this conversation. It is not the intention to bring up new points during this telephone conversation. Do you want to share your phone number?

Would you like to be kept informed about the research? I will note this, and you will receive updates via email. Thank you for your time and participation in this study.
